# MISEV2023 provides an updated and key reference for researchers studying the basic biology and applications of extracellular vesicles

**DOI:** 10.1093/stcltm/szae052

**Published:** 2024-07-19

**Authors:** Dinesh Upadhya, Ashok K Shetty

**Affiliations:** Centre for Molecular Neurosciences, Kasturba Medical College, Manipal, Manipal Academy of Higher Education, Manipal, 576104, India; Institute for Regenerative Medicine, Department of Cell Biology and Genetics, School of Medicine, Texas A&M University, College Station, TX 77843, United States

**Keywords:** extracellular vesicles, separation of extracellular vesicles, storage of extracellular vesicles, clinical application of extracellular vesicles

## Abstract

The recently published “Minimal information for studies of extracellular vesicles - 2023 (MISEV2023)” in the *Journal of Extracellular Vesicles* has provided practical solutions to the numerous challenges extracellular vesicles (EVs) researchers face. These guidelines are imperative for novice and experienced researchers and promote unity within the EV community. It is strongly recommended that laboratories working with EVs make MISEV2023 an essential handbook and that researchers actively promote these guidelines during laboratory meetings, journal clubs, seminars, workshops, and conferences. A collective effort from EV researchers is crucial to steer the progress of EV science in a positive direction.

**Significance Statement**

The “Perspective” discusses the recently published article “Minimal information for studies of extracellular vesicles—2023 (MISEV2023)” in the *Journal of Extracellular Vesicles*. This perspective recommends guidelines for both novice and experienced extracellular vesicles (EVs) researchers to promote unity within the EV community. It is also recommended that laboratories working with EVs make MISEV 2023 an essential handbook and that researchers actively promote these guidelines.

Research on extracellular vesicles (EVs) has continued to grow in the past 5 years. While new researchers entering the field of EVs are critical for continuing its development, they often face multiple challenges. These include navigating the complex terminology surrounding different types of EVs, such as exosomes, microvesicles, EVs, small EVs, large EVs, ectosomes, and extracellular particles. Additional critical issues include selecting an appropriate methodology for separating EVs that balances purity and yield, understanding the significance of size, morphology, and molecular characterization, and deciding the parental cells of EVs to employ in different studies and storage of EVs without losing their biological activity or therapeutic efficacy. The minimal information for studies of extracellular vesicles—2023 (MISEV2023), published recently in the *Journal of Extracellular Vesicles*, has addressed these challenges and provided practical solutions.^1^ Compared to MISEV2018,^2^ MISEV2023 presents an updated and comprehensive overview of various methods and their advantages and limitations for producing, isolating, and characterizing EVs from diverse sources. Additionally, it includes advanced techniques and approaches that are currently expanding the frontiers of the field, as well as new sections on EV release and uptake and in vivo methodologies for studying EVs.

The guidelines recommend using terms in EV research and their corresponding meanings, sources of EVs, and methods for separating, concentrating, characterizing, and storing them for preclinical, clinical, and research studies. The guidelines propose that researchers identify the rationale for attributing a function to EV components or a biomarker to EVs versus other components. Additionally, researchers are encouraged to report their methods in sufficient detail to enable replication of their studies. It is worth noting that MISEV23 is not a rigid checklist that dictates what should or should not be done but instead facilitates careful expert judgment in every experimental setup. MISEV2023 does not mandate using any particular marker or markers, enriched or depleted, nor does it prohibit any technique or platform. Furthermore, these guidelines are not barriers to innovation in EV research but instead suggest how innovative or new techniques can be presented and validated. It is also important to note that not following these guidelines does not necessarily mean that the publication or project will be rejected.

MISEV2023 has a separate section on nomenclature that provides all the essential information regarding using specific terminology for EVs.^1^ This section is easy for everyone to comprehend. EVs, nanosized vesicles released by cells delimited by a lipid bilayer, cannot replicate as they do not contain a functional nucleus. Operational terms such as small and large EVs are recommended to describe isolated vesicles from cells. Typically, small EVs are vesicles smaller than 200 nm in diameter, while large EVs are vesicles greater than 200 nm in diameter. However, it is essential to exercise prudence when using these terms, as the measured size can vary depending on the specific characterization method used by the investigator. Using the terms “exosomes” and “microvesicles” is highly discouraged for vesicles of any size. However, when the endosomal origin of the isolated particle is confirmed, the term “exosome” can be used.

Researchers interested in separating and storing EVs can access vast amounts of information.^1^ This information covers various topics such as the source of EVs, the quality and quantity of EVs, methodologies used to collect EVs, pre- and postpreparation handling, storage conditions, and pooling of EVs. While it is unrealistic to provide universal recommendations, specific recommendations are given based on available data for handling, processing, and storage conditions for EVs isolated from cell culture-conditioned medium, bacteria, blood, urine, cerebrospinal fluid, saliva, synovial fluid, milk, and solid tissues. These guidelines are valuable because using freshly isolated EVs for clinical applications is challenging.

Clinical applications of EVs are still in the early stages despite extensive preclinical studies testing their efficacy in models of human diseases. In 2021, the first human delivery of umbilical cord mesenchymal stem cell-derived EVs (MSC-EVs) was performed in a patient with Menière’s disease.^3^ The patient received EVs before undergoing cochlear implant surgery to attenuate the inflammation-based side effects of the procedure. At present, there are 73 clinical trials involving EVs, with MSC-EVs being used in 49 of them. Of these 49 trials, 25 are controlled clinical trials.^4^ While MSCs are the most commonly used parental cells for testing therapeutic EVs in various human disease models,^5,6^ preclinical studies are increasingly testing EVs derived from multiple sources, including neural stem cells, astrocytes, and microglia,^7-9^ and EVs carrying therapeutic compounds.^10^ Since EVs from a variety of stem cells and other postmitotic cells have the potential for therapeutic applications, studies employing the recommended guidelines in MISEV2023 for the separation and storage of EVs would reduce heterogeneity in the nucleic acid, protein, and lipid composition of EVs, thereby facilitating consistency in their therapeutic efficacy in preclinical and clinical trials. In addition, the publication of position statements and articles by scientific societies involved in EV research could significantly advance the field toward clinical applications. For instance, members of scientific societies have proposed specific harmonization criteria for MSC- and iPSC-derived small EVs to facilitate data sharing and comparison,^11-14^ representing a positive step in the right direction for the field.

EVs are isolated based on biophysical characteristics like size, density, charge, and specific surface molecules. Each separation method has unique advantages and limitations regarding purity and yield. Therefore, to select a suitable method for EV separation that balances purity and yield, a detailed section on “EV separation and concentration” is available. This section explains each separation method, including precipitation, differential ultracentrifugation, filter concentration, density gradient centrifugation, size exclusion chromatography, immunoprecipitation or affinity precipitation, and asymmetric flow field flow fractionation. It also provides a helpful graph of yield versus specificity for each method, which can help researchers choose a method that best suits their research requirements. Utilizing proprietary commercial kits, which safeguard their method’s principle and specific reagents, is strongly discouraged. The lack of transparency inherent in such kits can impede the replication and comparison of results across studies. In contrast, using a detailed and transparent protocol can ensure the reproducibility of the results. While combining complementary methods to increase yield and specificity is ideal, a researcher can choose a method that best suits their research requirement by analyzing its suitability over other methods.^15^ Such selection can be made by considering the plusses and shortcomings of each method in terms of purity and yield. Thus, when determining the optimal separation method, it is crucial to consider whether the primary objective is to maximize yield or ensure specificity. For instance, precipitation methods typically yield higher quantities but may lack specificity due to potential contamination with elements other than EVs. Conversely, size exclusion chromatography methods yield lower quantities but offer greater specificity for EVs. Precipitation methods remain favorable when dealing with limited sample volumes, given their higher yields. However, when larger sample volumes are available, size exclusion chromatography is preferred due to its enhanced specificity for EVs. A comprehensive understanding of each separation method is imperative for selecting the most appropriate approach for EV separation.

It is critical to accurately determine the quantity and identity of EVs and detect the presence of non-EV components in EV preparations. Standard techniques such as nanoparticle tracking analysis (NTA), dynamic light scattering (DLS), flow cytometry, or resistive pulse sensing (RPS) could be used to identify concentration and size. However, MISEV2023 guidelines also support using different physical principles of EVs to minimize method-specific biases and EV particle size distribution. Additionally, the document provides clear and concise recommendations for quantifying total EV protein, lipids, and RNA analysis. Regular fluorescence microscopy is diffraction-limited, so different approaches are needed to characterize small particles. However, for smaller-sized EVs, scanning electron microscopy (EM), transmission EM, cryo-EM, and scanning-probe microscopy, including atomic force microscopy, must be used.

MISEV2023 has recommended a comprehensive 5-component framework to report the protein content of EVs. This framework follows the 2018 guidelines^2^ and includes multiple categories that assess the presence, purity, and possible contamination of EVs. Using this framework, researchers can accurately evaluate EV preparations for membrane-associated proteins (category 1) and cytosolic proteins (category 2), common contaminants such as lipoproteins, proteins, and nucleic acid aggregates (category 3), and intracellular components, including those from the nucleus, mitochondria, endoplasmic reticulum and Golgi apparatus (category 4). Researchers are also recommended to assess co-isolates with EVs, such as secretory proteins (category 5).

To ensure accurate and reliable EV characterization, MISEV2023 recommends technique-specific reporting considerations. Recommendations are available for flow cytometry (including bead-based and single EV flow cytometry), genetic protein tagging, mass spectrometry proteomics, microscopy (atomic force, diffraction-limited fluorescence, DLS, EM), NTA, single-particle interferometric reflectance imaging sensing and super-resolution microscopy, nucleic acid characterization, protein and nonprotein labeling, Raman spectroscopy, RPS, and Western blotting. By following these guidelines, researchers can improve the reproducibility of their findings in EV research.

MISEV2023 also addressed several other aspects, such as recommendations for the release and uptake of EVs, functional in vitro studies using EVs, and analyzing them under in vivo conditions. Given the vast array of functional studies conducted in vivo and in vitro, the guidelines only provide general recommendations. Authors are encouraged to employ and report innovative approaches in various organisms to advance the field.

In summary, the MISEV2023 guidelines offer valuable guidance for novice and seasoned EV researchers while promoting a sense of solidarity within the EV community. By joining forces, experts in the field can deepen our understanding of the role of EVs in crosstalk between different types of cells and organs in physiological conditions, the pathophysiology and progression of various human diseases, and leverage their potential for clinical applications that can improve human health. It is highly recommended that laboratories working with EVs make MISEV2023 an essential handbook and that researchers promote awareness of these guidelines during laboratory meetings, journal clubs, seminars, workshops, and conferences. A collective commitment from EV researchers can help steer the course of EV science in a positive direction. Finally, it is to be noted that MISEV2023 presents the current best practice in the field and reflects the current consensus position of the EV community. However, it does not impede the development of newer and advanced techniques.

## Data Availability

All data needed to evaluate the conclusions of this perspective are present in the article.
